# Correction to: Capturing Data on Antimicrobial Resistance Patterns and Trends in Use in Regions of Asia (CAPTURA) Supplement

**DOI:** 10.1093/cid/ciae122

**Published:** 2024-03-29

After the original publication of this supplement, [Capturing Data on Antimicrobial Resistance Patterns and Trends in Use in Regions of Asia (CAPTURA), *Clinical Infectious Diseases* Volume 77 Issue Supplement_7] there were several required updates to be made in three of the articles appearing here. Below are the three articles updated post-publication and a summary of their changes.

*Clinical Infectious Diseases*, Volume 77, Issue Supplement_7, 15 December 2023, Pages S528–S535, https://doi.org/10.1093/cid/ciad667. The Acknowledgments section of this article has been updated with specific organization details for appreciation.

*Clinical Infectious Diseases*, Volume 77, Issue Supplement_7, 15 December 2023, Pages S500–S506, https://doi.org/10.1093/cid/ciad567. Several authors in the byline of this article had erroneously inserted middle initials. These have been removed in the version currently available online.

*Clinical Infectious Diseases*, Volume 77, Issue Supplement_7, 15 December 2023, Pages S569–S577, https://doi.org/10.1093/cid/ciad647. In this article the author Zahra Lakdawala's affiliation was originally incorrect and has been updated in the version currently available online.

